# Manual Dexterity is not Related to Media Viewing but is Related to Perceptual Bias in School-Age Children

**DOI:** 10.3390/brainsci10020100

**Published:** 2020-02-13

**Authors:** Satoshi Nobusako, Taeko Tsujimoto, Ayami Sakai, Takashi Shuto, Emi Furukawa, Michihiro Osumi, Akio Nakai, Takaki Maeda, Shu Morioka

**Affiliations:** 1 Neurorehabilitation Research Center, Kio University, Nara 635-0832, Japan; 2 Graduate School of Health Science, Kio University, Nara 635-0832, Japan; 3 Department of Rehabilitation, Nishide Clinic, Osaka 532-0002, Japan; 4 Department of Rehabilitation, Higashi Osaka Yamaji Hospital, Osaka 578-0925, Japan; 5 Department of Home-Visit Rehabilitation, Ishida Clinic, Osaka 592-0002, Japan; 6 Graduate School of Clinical Education & The Center for the Study of Child Development, Institute for Education, Mukogawa Women’s University, Hyogo 663-8558, Japan; 7 Department of Neuropsychiatry, Keio University School of Medicine, Tokyo 160-8582, Japan

**Keywords:** manual dexterity, media preference level, media viewing time, perceptual bias, school-age children, temporal order judgment (TOJ) task

## Abstract

Although the media can have both negative and positive effects on children’s cognitive and motor functions, its influence on their perceptual bias and manual dexterity is unclear. Thus, we investigated the association between media viewing time, media preference level, perceptual bias, and manual dexterity in 100 school-aged children. Questionnaires completed by children and their parents were used to ascertain media viewing time and preference levels. Perceptual bias and manual dexterity were measured using the visual-tactile temporal order judgment task and Movement Assessment Battery for Children—2nd edition, respectively. There were significant positive correlations between age and media viewing time and between media viewing time and media preference level. There was also a significant negative correlation between visual bias and manual dexterity. Hierarchical multiple regression analysis revealed that increasing visual bias was a significant predictor of decreasing manual dexterity. Further, children with low manual dexterity showed significant visual bias compared to those with high manual dexterity, when matched for age and gender. The present results demonstrated that, in school-aged children, although viewing media was not associated with perceptual bias and manual dexterity, there was a significant association between perceptual bias and manual dexterity.

## 1. Introduction

Studies have suggested that media can have both positive and negative effects on health, cognitive abilities, and motor function in children [1,2,3,4]. The term media includes broadcast media, such as TVs and movies, and interactive media, such as social media and video games, which allow users to consume and create content [1,2,3,4]. The former is passive media, while the latter is active media.

In children, time spent watching TV, DVDs/videos, and internet content is associated with poor health conditions such as obesity [5,6,7,8,9,10] and sleep disorders [11,12,13,14,15]. In addition, media viewing time has been shown to have a negative effect on cognitive functions such as delayed language development [16,17] and decreased attention [18]. Zimmerman et al. [16] demonstrated that media viewing in infants leads to poor language development. Dworak et al. [17] suggested that, even in school-age children, media viewing adversely affects sleep and decreases verbal cognitive performance. Further, Christakis et al. [18] reported that media viewing at the age of 1–3 years was associated with attention problems at the age of 7 years. Similarly, there is evidence of the negative impact actively playing video games can have on the cognitive skills of children. Increased video game play time is also associated with attentional problems in children as reported by parents and teachers [19], as well as decreased verbal cognitive performance, and problems with memory, learning, and sleep [17]. In particular, media viewing in children under the age of 2 years has a negative effect on children’s cognitive development (language, executive function) [4,20,21].

In addition, the displacement hypothesis states that the time children spend using media limits the time they have to do other activities, which can lead to a decrease in physical activity [22,23,24]. Thus, media viewing may displace sensorimotor experiences (e.g., manipulation, climbing) with the development of visuomotor skills [25]. In addition, Lin et al. [26] reported that preschool children who did not use a touch screen tablet had improved manual dexterity compared to preschool children who did use the touch screen tablet, which indicated that using interactive media might be disadvantageous for the development of manual dexterity of preschool children [26]. Thus, previous studies have shown that media viewing can have various negative effects on cognitive and motor functions in children.

Conversely, however, there is also evidence that media viewing positively affects physical motor function and cognitive functions such as language, attention, and executive function in children. Preschool educational television programs were reported to have a positive impact on linguistic skills such as vocabulary and literacy in children [27]. Educational television viewing was also associated with greater school readiness and increased academic performance that can be traced through high school [28,29]. Neuman [30] showed that preschool children who used touch screen tablets had improved language skills such as letter name, sound knowledge, print concepts, and name writing compared to preschool children who did not use them. Stevens and Mulsow [31] reported that media viewing during preschool was not related to attention issues such as attention-deficit/hyperactivity disorder during the first grade. Foster and Watkins [32] investigated the association between media viewing at 1–3 years of age and attentional issues at 7 years of age and found that there was no significant relationship between television viewing and attentional issues. In addition, Zimmerman and Christakis [33] reported that educational television viewing under the age of 3 years was not associated with attention problems after 5 years. Linebarger et al. [34] showed that parenting style and educational television exposure alleviated the negative effect on the executive function of media viewing in preschool and school-aged children.

Several studies have found an association between using interactive media, such as a touch screen tablet, and improving manual dexterity in 2–3 year olds [35,36,37]. Bedford et al. [35] showed that there was a significant correlation between the age of first touchscreen use and the achievement of fine motor milestones and suggested that infants who actively use a touchscreen earlier also develop earlier manual dexterity abilities. In addition, interventions using interactive media such as video games have been shown to be effective in improving childhood movement disorders such as cerebral palsy [38] and developmental coordination disorder [39].

Thus, viewing media can have both negative and positive effects on the cognitive and motor functions of children. However, many factors, including child age, parenting style, type of content (educational media or not), socioeconomic status, coviewing with a parent, and child temperament, can modulate media viewing and affect cognition and motor functions in children [25]. 

However, few studies have investigated in detail how media viewing habits affect other cognitive functions in children. The influence of media viewing habits on children’s visual bias in particular is not clear. Here, visual bias alludes to the focus on visual information when visual and tactile stimuli are given almost simultaneously [40], rather than giving tactile information important for manual dexterity in isolation [41,42,43,44]. In addition, although studies have examined the effects of using interactive media such as a touch screen tablet on children’s manual dexterity [26,35,36,37], the effects of general media viewing, including passive media, on manual dexterity have not been clarified in children. In addition, an increase in visual bias has been shown to lead to a decrease in manual dexterity in children with motor impairment [45,46]. However, the relationship between visual bias and manual dexterity in children with typical development has not been clarified. Frequent exposure of media may increase perceived visual bias and, consequently, decrease perceived tactile bias. In addition, considering the important relationship between tactile sensation, visual bias, and manual dexterity shown by previous studies [41,42,43,44,45,46], an increase in visual bias may lead to a decrease in manual dexterity, even in children with typical development.

Therefore, an increase in media viewing in children may increase attention and sensitivity to visual information and increase their bias towards visual information, resulting in a decrease in manual dexterity. To test this hypothesis, we conducted questionnaires on media viewing and measured perceptual bias and manual dexterity of school-aged children. The questionnaires on media viewing included the average viewing time per day and preference level for media in children. We also used the visuo-tactile temporal order judgment task as a quantitative measure of perceptual bias in the current study. The temporal order judgment paradigm enabled characterization of biases indicated by the shift of the judgment to the advantage of one of the two stimuli and quantitative measurement of the effects of perceptual bias [40,47,48]. We used the manual dexterity test of the Movement Assessment Battery for Children—2nd edition [49], which is an international standard evaluation battery of motor function in children, to measure manual dexterity in the current cohort. Correlation and multiple regression analyses were performed to examine the relationships between these variables.

## 2. Materials and Methods

### 2.1. Participants

A total of 100 school-aged children (mean age ± standard deviation (SD), 9.2 ± 1.9 years; range, 6–12 years; 40 male participants; 87 right-handed) enrolled in regular classes at public primary schools participated in the current study. Children with typical development who were enrolled in regular classes at public primary schools in Osaka and Nara, Japan, were recruited for this study. The exclusion criteria consisted of the following: (1) a general medical condition (e.g., cerebral palsy, hemiplegia, and muscular dystrophy), (2) diagnosis of a developmental disorder (e.g., autism spectrum disorder, attention deficit hyperactivity disorder, developmental coordination disorder, learning disorder, and pervasive developmental disorder), or (3) diagnosis of intellectual disability. Eligibility was confirmed by interviewing parents and the results of regular checkups, which were provided by the school doctor at each school.

All experimental procedures were approved by the local ethics committee of the Graduate School and Faculty of Health Sciences at Kio University (approval number: R1-22). There were no foreseeable risks, and no personally identifying information was collected. The participants (children and their parents) provided background information and written informed consent. The procedures complied with the ethical standards of the 1964 Declaration of Helsinki regarding the treatment of human participants in research. Table 1 summarizes the participants’ age, sex, and preferred hand distributions.

### 2.2. Procedures

All participating children completed the media viewing questionnaire, visual-tactile temporal order judgment (TOJ) task, and manual dexterity test of the Movement Assessment Battery for Children—2nd edition (M-ABC-2). The questionnaire and two experimental tasks were performed in random order for each child. The questionnaire, TOJ task, and manual dexterity test took less than 10, 20, and 30 minutes to complete, respectively. Therefore, each child completed all trials within 1 hour.

#### 2.2.1. Questionnaire on Media Viewing

The children and their parents answered the questionnaire on media viewing. Media here included TV, DVD, internet content, such as YouTube and Netflix, and various video games. Media provision equipment included TVs, PCs, tablets, smartphones, and game devices such as a PlayStation and Nintendo Switch. There were two questions in the media viewing questionnaire.

Question 1: The average time per day for children’s media viewing (as an average for weekdays, weekends, and holidays). This question did not include parents’ media viewing time and was answered as a single answer. Possible answers included the following: 0, 1, 2, 3, 4, and 5 hours or more. These answers were converted to 0, 1, 2, 3, 4, and 5 points, respectively [50,51,52].

Question 2: Preferred media level, which was answered by the children alone. Media preference level was measured using the following 7-item scale: 3, I like the media very much.; 2, I like the media.; 1, I like the media a bit.; 0, I do not like or hate the media (It is neither).; −1, I hate the media a bit.; −2, I hate the media.; −3, I hate the media very much.

#### 2.2.2. Temporal Order Judgment Task

**Figure 1:**
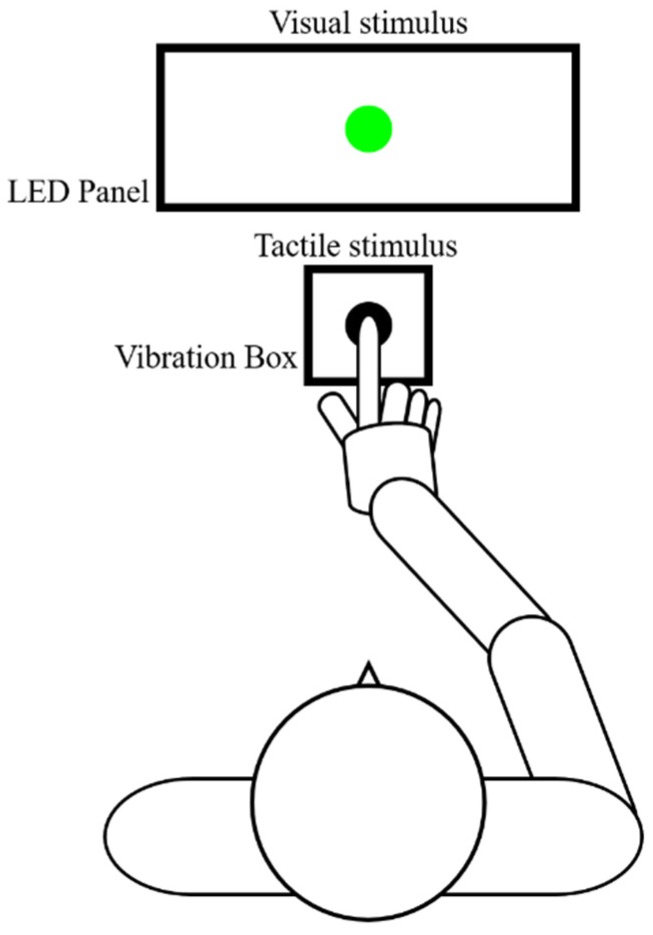
Temporal order judgment (TOJ) task. The task was presented to the child with a visuo-tactile TOJ device (Keio method, UT-0021, Medical Try System, Tokyo, Japan). This device includes an LED panel (UT-0021-2, Medical Try System, Tokyo, Japan) and vibration box (UT-0021-1, Medical Try System, Tokyo, Japan), which provided the visual and tactile stimuli, respectively. The child put the index finger of their preferred hand in the hole of the vibration box and touched the vibrotactile stimulator. Therefore, the child could not observe the tactile stimulus. The child was instructed to watch the LED panel. Conditions were set in the TOJ task device so that the visual stimulus was presented 0, 50 or 100 ms earlier than the tactile stimulus, or vice versa. A blackout curtain prevented the child from seeing outside the experimental chamber.

Perceptual biases were measured using the TOJ task [53,54,55,56,57] (Figure 1). Two stimuli (visual-flash and tactile-vibration) were presented in various stimulus onset asynchronies (SOAs). The child then had to determine which stimulus (visual or tactile) was presented first. The child performed this visuo-tactile TOJ task with a TOJ task device (Keio method, UT-0021, Medical Try System, Tokyo, Japan). The visual stimulus was presented as a green LED on the LED panel (UT-0021-2, Medical Try System, Tokyo, Japan). The luminance of the visual stimulus was 40 cd/m^2^, and the duration of the visual stimulus was 1 ms. A 1-ms tactile stimulus (converted to vibration by pneumatic pressure) controlled by a 1-V signal from the vibration box (UT-0021-1, Medical Try System, Tokyo, Japan) was presented to the preferred index finger. 

The stimulation condition included the following five conditions: at −100, −50, 0, 50, 100 ms, i.e., tactile stimulation was administered 50 or 100 ms earlier than the other in the −100 and −50 ms conditions, while visual stimulation was administered first in the 50 and 100 ms conditions; visual and tactile stimulation was synchronous in the 0 ms condition. In the actual test, the five stimulation conditions were considered a set, and each child performed five sets; the trial order was randomized. Therefore, each child completed 25 trials in total. 

Before starting the TOJ task, simple stimulus tests were used to confirm that children had no problems with vision and touch. Specifically, the tactile and visual stimuli used for the TOJ task were administered five times alone in the absence of the other stimuli to ascertain whether all the children were able to perceive the tactile and visual stimuli. The TOJ task was conducted after sufficient explanation and practice were provided to the child.

The “visual first” response probability for each of several SOA conditions (−100, −50, 0, 50, 100) was then calculated for the TOJ task. The following formula was used to fit the logistic curves to the “visual first” response probability in the TOJ task [58,59,60]:

$$P\left(t\right)\;=\;\frac{1}{1+\exp\left(-a\left(t-t_{PSE}\right)\right)}$$

where *t* is the SOA, *P*(*t*) is the probability of the “visual first” response, a is the steepness of the fitted curve, and tPSE is the observer’s point of subjective equality (PSE), the last of which demonstrates the SOA where “visual first” and “tactile first” judgment probabilities are equal (50%). A nonlinear least squares algorithm was used to fit the data in MATLAB R2014b (MathWorks, MA, USA). The PSE of each child is a quantitative indicator of perceptual bias of the individual; a large negative PSE value indicates that visual bias is strong, and a large positive PSE value indicates that tactile bias is strong. Therefore, a PSE value approaching 0 ms demonstrated no biased perception.

#### 2.2.3. Manual Dexterity Test of the Movement Assessment Battery for Children—2nd Edition (M-ABC-2)

The manual dexterity test of the M-ABC-2 [49] is a standardized, age-adjusted test to identify motor problems in children, in which different tasks are administered to children in different age bands. The M-ABC-2 has good test retest reliability (minimum value at any age was 0.75), inter-rater value (0.70), and concurrent validity [49]. This test has the following three age bands: 3–6, 7–10, and 11–16 years. 

Our study included children aged 6 to 12 years of age. Each child took three tests that were appropriate for their age band. The 6-year-old children were in age band 1 and were administered the following three tests: posting coins test, threading beads test, and drawing trail I test. The children aged 7–10 years old were in age band 2 and were administered the following tests: placing pegs test, threading lace test, and drawing trail II test. The children aged 11–12 years old were in age band 3 and were administered the following tests: turning pegs test, triangle with nuts and bolts test, and drawing trail III test. According to the examiner’s manual of M-ABC-2, standard scores of the participants are calculated from the obtained raw scores. The standard score reflects the degree of manual dexterity for each year of age, in which a higher standard score represents improvement of manual dexterity within each age group. A specifically trained and certified physical therapist administered all of these assessments.

### 2.3. Statistical Analyses

All statistical analyses were performed using SPSS ver. 26 (SPSS, Chicago, IL, USA). Age, media viewing time, media preference level, perceptual bias, and manual dexterity were analyzed using correlation and hierarchical multiple regression analyses. In addition, between-group comparisons based on manual dexterity scores were also performed.

#### 2.3.1. Correlation Analysis

Since age, media viewing time, media preference level, perceptual bias, and manual dexterity data were not normally distributed, data were analyzed using a Spearman’s correlation coefficient by rank test. The level of statistical significance was set at *p* < 0.05.

#### 2.3.2. Hierarchical Multiple Regression Analysis

In the current study, we hypothesized that an increase in media viewing would increase visual bias and eventually reduce manual dexterity. Therefore, we performed hierarchical multiple regression analysis (forced entry method) with manual dexterity as the dependent variable and age, media viewing time, media preference level, perceptual bias, and interaction terms as independent variables. We created three interaction terms, considering the possibility that the media viewing time and the media preference level have become moderator variables, which may enhance the relationship between the increase in visual bias and the decrease in manual dexterity. These were interaction term-1 (perceptual bias × media viewing time), interaction term-2 (perceptual bias × media viewing level), and interaction term-3 (perceptual bias × media viewing time × media preference level). The interaction terms were calculated by multiplying the values obtained by centering each variable, taking into account the multicollinearity. In model 1, age, media viewing time, media preference level, and perceptual bias were independent variables. In model 2, interaction term-1 (perceptual bias × media viewing time) was added to model 1 as an independent variable. In model 3, interaction term-2 (perceptual bias × media preference level) was added to model 2 as an independent variable. In model 4, interaction term-3 (perceptual bias × media viewing time × media preference level) was added to model 3 as an independent variable. The statistical significance level was set at *p* < 0.05.

#### 2.3.3. Comparison between Groups

Since children in the 63rd percentile (standard manual dexterity test score, 11) or higher have relatively greater manual dexterity ability, they were classified as the ‘high manual dexterity group’. In contrast, children in the 50th percentile (standard manual dexterity score, 10) or lower were classified as the ‘low manual dexterity group’. Gender and preferred hand between the two groups were compared using a chi-squared test for independence. Data (age, media viewing time, media preference level, and perceptual biases) of both groups were compared using a Mann-Whitney U test since data were not normally distributed. The level of statistical significance was set at *p* < 0.05. The effect size was also calculated [61].

## 3. Results

Table 2 shows a summary of all data obtained from 100 children. The raw data of all participants are shown in Table A1.

### 3.1. Correlation Analysis Results

**Figure 2:**
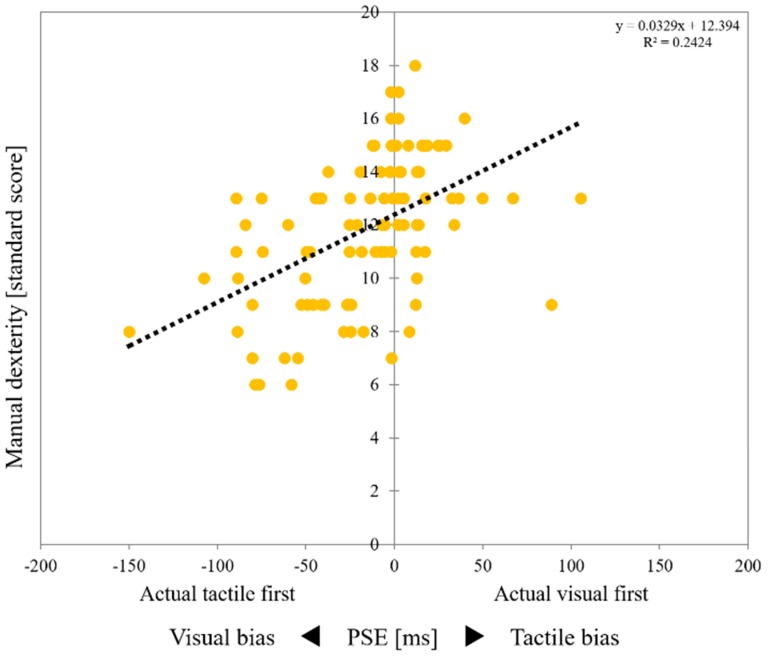
Correlation between perceptual bias and manual dexterity. Scatter plot showing the relationship between the temporal order judgment task and manual dexterity test. The vertical axis shows the results of the manual dexterity test (standard score) of each child. An increase in the standard score indicates an improvement in manual dexterity. The horizontal axis shows the results of the temporal order judgment task (PSE) for each child. Negative PSE values on the horizontal axis represent “visual first” indicators that were higher although the tactile stimulus was actually faster than the visual stimulus. Thus, an increase in the negative PSE value represents an increase in visual bias. Conversely, positive PSE values on the horizontal axis represent the “visual first” indicators when the visual stimulus was actually earlier than the tactile stimulus. Thus, an increase in the positive PSE value represents an increase in tactile bias. Therefore, the closer the PSE value is to 0 (middle line), the lower the perception bias. PSE, point of subjective equality.

Table 3 outlines the results of the correlation analysis. There was a significant correlation between age (years) and media viewing time (hour, Questionnaire 1) (*p* = 0.003, r = 0.293) (Table 3). There was also a significant correlation between media viewing time (hour, Questionnaire 1) and media preference level (Questionnaire 2) (*p* = 0.007, r = 0.269) (Table 3). Further, there was a significant correlation between perceptual biases (ms, PSE) and manual dexterity (standard score) (*p* < 0.001, r = 0.537; Table 3, Figure 2). However, there was no significant correlations between age, media viewing time, and media preference level and perceptual biases or manual dexterity (Table 3).

### 3.2. Hierarchical Multiple Regression Analysis Results

The results of the hierarchical multiple regression analysis are summarized in Table 4. The results of the hierarchical multiple regression analysis showed that model 1 had the best fit compared to other models (model 2–4). For manual dexterity, compared to the other models (model 2–4), model 1 showed the highest coefficient of determination adjusted for the degrees of freedom (adjusted R2), a significant change in the multiple coefficient of determination (R2), and the lowest Akaike information criterion (AIC) and Bayesian information criterion (BIC), indicating that model 1 was the best fit for the data. In addition, there was no interaction effect between perceptual bias and media viewing/preference level. Therefore, the media viewing time/preference level was not a moderator variable that strengthened the relationship between increasing visual bias and decreasing manual dexterity.

The detailed results of the hierarchical multiple regression analysis (model 1) are as follows: with manual dexterity as a dependent variable, only perceptual bias was a significant independent variable (β = 0.501, *p* < 0.001). Age, media viewing time, and media preference level were not significant independent variables of manual dexterity. The relationship between manual dexterity and perceptual bias could be modeled with the following equation: Manual dexterity = 13.080 + (0.033 × perceptual bias), resulting in the following results: R = 0.503, *R*^2^ = 0.253, Adjusted *R*^2^ = 0.221, *p* < 0.001. In addition, there was no multicollinearity effect (Table 4).

### 3.3. Comparison Results between Groups

As a result of grouping based on the manual dexterity test score, 71 children (25 male participants; 63 right-handed) and 29 children (15 male participants; 24 right-handed) were grouped into high and low manual dexterity groups, respectively. Table 5 summarizes the data for each group. There was no significant difference in sex (χ^2^(0.95) = 3.841, χ^2^ = 2.339, *p* = 0.126) and preferred hand (χ^2^(0.95) = 3.841, χ^2^ = 0.650, *p* = 0.420) between groups. Further, there were no significant differences in age (*z* = −0.284, p = 0.776, effect size (r) = −0.03), media viewing time (*z* = −0.474, *p* = 0.635, effect size (r) = −0.05), and media preference level (*z* = −0.051, *p* = 0.959, effect size (r) = −0.01) among the groups.

**Figure 3:**
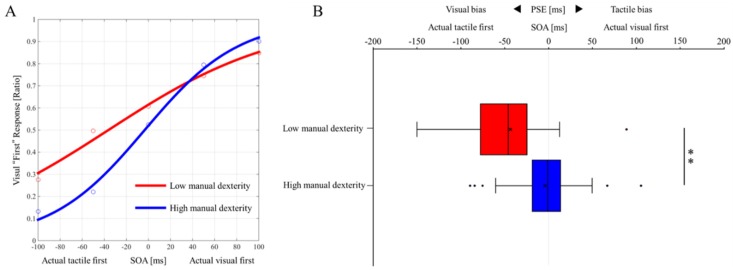
The probability curve of the “visual first” response in each group and the inter-group comparison results of perceptual biases. (**A**) The "visual first" response probability curves of each group in the TOJ task. Blue, high manual dexterity children group (*n* = 71, mean ± standard deviation, −4.3 ± 33.8). Red, low manual dexterity children group (*n* = 29, mean ± standard deviation, −43.4 ± 44.0). (**B**) The comparison results of perceptual biases (PSE) between groups. Blue box, high manual dexterity children group (*n* = 71). Red box, low manual dexterity children group (*n* = 29). Lines represent the range of the minimum (left end) and maximum (right end). Boxes represent the lower (left end), median (center line), and upper (right end) quartiles. Low manual dexterity children group: maximum = 88.84, upper quartile = −24.72, median quartile = −45.87, lower quartile = −76.54, minimum = −150. High manual dexterity children group: maximum = 105.4, upper quartile = 13.22, median quartile = −1.004, lower quartile = −16.25, minimum = −89.47. ** *P* < 0.01; * *P* < 0.05. TOJ, temporal order judgement; PSE, point of subjective equality; SOA, several stimulus onset asynchronies.

Figure 3A shows the “visual first” response probability curves for both groups, and Figure 3B shows the perceptual biases (PSE) comparison results for both groups. The PSE of the low manual dexterity group (mean ± standard deviation, −43.4 ± 44.0) was significantly lower than the PSE in the high manual dexterity group (mean ± standard deviation, −4.3 ± 33.8) (*z* = −4.543, *p* < 0.001, effect size (r) = −0.45; Figure 3B). This indicated that the low manual dexterity group had a significantly stronger visual bias than the high manual dexterity group.

## 4. Discussion

The current study analyzed the association between media viewing time, media preference level, perceptual bias, and manual dexterity in school-aged children (6–12 years). There were significant correlations between age and media viewing time and between media viewing time and media preference level. This indicated that children growing older is associated with an extension in media viewing time, which in turn is related to an increased preference for media. However, there was no significant correlation between media viewing time and/or media preference level and perceptual bias and/or manual dexterity. Conversely, there was a significant correlation between the increase in visual perceptual bias and the decrease in manual dexterity. Furthermore, the results of hierarchical multiple regression analysis showed that among the variables (i.e., age, media viewing time, media preference level, and perceptual bias), only perceptual bias was a significant independent variable for manual dexterity. Media viewing time and media preference level did not strengthen the relationship between increasing visual bias and decreasing manual dexterity. In addition, children with low manual dexterity showed significant visual bias compared to those with high manual dexterity.

The significant correlation between age and media viewing time is consistent with previous studies, which have reported this association in children aged <3 years and 3–16 years old [35,51,62,63,64,65,66,67,68]. The current study also demonstrated a significant correlation between media viewing time and media preference level. 

However, there was no significant correlations between media viewing time and/or media preference level and perceptual bias and/or manual dexterity in the current study. In addition, hierarchical multiple regression analysis did not reveal the effect of media viewing on perceptual bias and manual dexterity. Previously, various adverse effects of media viewing on cognitive functions and motor functions in children, such as language development delay [16,17], poor health [9,22], and attention problems [18], have been reported. However, these effects may be mitigated by factors such as parenting style [34], type of content and program [69], and co-viewing with a parent [70]. This could be attributed to the variation in parenting styles, i.e., authoritative or permissive parenting based on Baumrind’s conceptualization [71], where the style of parenting determines the degree of discipline and the children’s responsiveness. Further, educational internet content and TV programs can have a positive impact on cognitive development in children [4]. In addition, socioeconomic status, such as education, income and deprivation, is associated with children’s media viewing and health status. Many studies have found an increase in media viewing time and sedentary time as the socioeconomic status decreases [72,73,74,75,76,77,78]. An increase in sedentary time can lead to a decrease in the time spent engaging in play and sports activities, which in turn can reduce manual dexterity. However, since we did not collect data on parenting style, type of content and program, and socioeconomic status, the effects of these factors in the current study are completely speculative. 

There is also some evidence that active video game enhances cognitive functions, such as visual processing and attention, and contributes to the improvement of motor control [79]. Thus, despite the negative risks, media can also have positive effects on children. In addition to video games that required movement, media included in the current study also included TV and DVDs/videos, which only required passive viewing. Therefore, our findings may have been different if media were classified into interactive and broadcast media, which are active and passive, respectively.

This is also supported several studies investigating the relationship between the use of a touch screen tablet, which can be manipulated and drawn on by touching the screen, and manual dexterity. Lin et al. [26] revealed that manual dexterity improves in children who did not use tablets compared with those who did. This was attributed to the fact that real actions of grasping, drawing, and manipulating objects required more muscle power, coordination, and dexterity than the virtual actions on a tablet [26]. However, other studies have reported discrepant results to Lin et al. [26]. Several previous studies demonstrated that the use of tablets is significantly associated with an improvement in manual dexterity, emphasizing the similarities between the virtual environment on the tablet screen and the real physical environment [35,36,37]. Therefore, the type of media used also seems to affect manual dexterity. Since we did not differentiate the type of media in the current study, it is not easy to discern whether this would have affected perceptual bias/manual dexterity. However, the relationship between media viewing time/media preference level and perceptual bias/manual dexterity should be explored in further future studies considering the limitations of the current study.

Importantly, the current study demonstrated a significant correlation between the increase in visual bias and decrease in manual dexterity in school-aged children with typical development. Further, increased visual bias was a significant predictor of reduced manual dexterity. In addition, participants with low manual dexterity showed significant visual bias compared to those with high manual dexterity. It is important to note that the children in the current study had typical development and had neither visual nor tactile problems when completing the simple stimulus tests prior to the TOJ task. Therefore, the current results could not be attributed to visual or tactile disorders. The tactile sensation of the hand is a prerequisite for manual dexterity, such as grasping objects, manipulating objects, and handwriting [41,42,43,44]. A study demonstrated that adult string players have an expanded cortical representation of somatosensory sensations of the fingers [80], which is direct evidence of the important relationship between manual dexterity and somatosensory sensation. Further, preschool children with motor delays show a significant correlation between tactile sense and fine motor skills [81]. Further, children with cerebral palsy [82] and Duchenne muscular dystrophy [83] show a significant relationship between hand tactile function and manual dexterity. Previous studies have also demonstrated that children with clumsy movements reported relying on visual information, and not tactile information, for hand movements [45,46]. Several previous studies have suggested that prioritizing visual information, not tactile information that is important for movement, adversely affects the success of the movement task [45,46,84,85,86,87,88,89]. Both visual and tactile information are important resources for manual dexterity. However, even if there is no visual information, manual dexterity can be completed with tactile information only. Therefore, the present results showed that prioritizing visual information over tactile information when visual and tactile stimuli are input almost simultaneously leads to poor manual dexterity, even in school-aged children with typical development.

### Limitations of the Current Study and Future Directions

Since the current study did not investigate the type of media (i.e., broadcast media or interactive media, active media or passive media, etc.), type of content (educational or otherwise), parenting style or socioeconomic status, we cannot determine whether these factors influenced the results in the current study. These factors play an important role in understanding the effect of media viewing on children’s cognitive and motor functions. Thus, further research, which includes measures of these factors, is required to better understand the relationship between media viewing and perception bias/manual dexterity in children.

All children who participated in the current study could detect a 1-ms tactile stimulus controlled by a 1-V signal in the TOJ task. However, the children’s tactile threshold (tactile sensitivity) was not measured. Therefore, differences in tactile sensitivity may have affected the results of the manual dexterity test. Taking this limitation into account, future studies will also require measurement of tactile sensitivity.

## 5. Conclusions

There was no significant correlation between media viewing time and/or media preference level, and perceptual bias and/or manual dexterity in school-aged children with typical development. However, there was a significant association between age and media viewing time and between media viewing time and media preference level in the current cohort. Further, there was also a significant correlation between visual bias and manual dexterity. Finally, increased visual bias was a significant predictor of reduced manual dexterity, which indicated that children with relatively low manual dexterity had strong visual bias.

## Figures and Tables

**Table 1:** Distributions of age, sex, and preferred hand of the entire cohort.

Age (years)	Number	Sex		Preferred Hand (Handedness)	
		Male (*n*)	Female (*n*)	Right (*n*)	Left (*n*)
6	8	3	5	8	0
7	16	6	10	13	3
8	17	5	12	12	5
9	14	7	7	11	3
10	16	7	9	14	2
11	12	6	6	12	0
12	17	6	11	17	0
Total	100	40	60	87	13

*n* = number.

**Table 2:** Summary of collected data.

*N* = 100	Age (years)	Media Viewing Time (hour)	Media Preference Level	Perceptual Biases (PSE; ms)	Manual Dexterity (Standard Score)
Mean	9.2	2.4	2.4	−15.6	11.9
Standard deviation	1.9	1.2	0.8	41.1	2.7
Range	6–12	0–5	0–3	−150–105.4	6–18
Skewness	0.02	0.54	−1.20	−0.31	−0.25
Kurtosis	−1.20	−0.25	0.59	0.99	−0.63

PSE, point of subjective equality.

**Table 3:** Correlation matrix data.

	Age (years)	Media Viewing Time (hour)	Media Preference Level	Perceptual Biases (PSE; ms)	Manual Dexterity (Standard Score)
Age (years)	-	0.293 **	−0.077	−0.016	−0.013
Media viewing time (hour)		-	0.269 **	−0.100	0.011
Media preference level			-	−0.101	−0.052
Perceptual biases (PSE; ms)				-	0.537 **
Manual dexterity (standard score)					-

** *p* < 0.01; *N* = 100. Numbers in the frame show correlation coefficients. PSE, point of subjective equality.

**Table 4:** Hierarchical multiple regression analysis results.

Dependent Variable	Model	Independent Variable	Partial Regression Coefficient (B)	Standardized Regression Coefficient (β)	*p*-Value	VIF	AIC	BIC
Manual dexterity	1	(constant)	13.080		<0.001		182.715	195.741
		Age	−0.092	−0.064	0.499	1.139		
		Media viewing time	0.236	0.108	0.285	1.268		
		Media preference level	−0.169	−0.052	0.585	1.138		
		Perceptual biases	0.033	0.501	<0.001	1.011		
		R = 0.503, *R*^2^ = 0.253, Adjusted *R*^2^ = 0.221, *p* < 0.001; Δ*R*^2^ = 0.253, ΔF = 8.027, *p* < 0.001						
	2	(constant)	13.081		<0.001		184.715	200.346
		Age	−0.092	−0.064	0.502	1.143		
		Media viewing time	0.236	0.108	0.287	1.268		
		Media preference level	−0.169	−0.052	0.587	1.139		
		Perceptual biases	0.033	0.501	<0.001	1.034		
		Interaction effect 1	0.000	0.000	0.999	1.027		
		R = 0.503, *R*^2^ = 0.253, Adjusted *R*^2^ = 0.213, *p* < 0.001; Δ*R*^2^ < 0.001, ΔF < 0.001, *p* = 0.999						
	3	(constant)	13.158		<0.001		186.005	204.242
		Age	−0.094	−0.066	0.491	1.143		
		Media viewing time	0.240	0.109	0.281	1.269		
		Media preference level	−0.189	−0.058	0.545	1.146		
		Perceptual biases	0.035	0.518	<0.001	1.089		
		Interaction effect 1	0.002	0.027	0.777	1.169		
		Interaction effect 2	−0.006	−0.079	0.418	1.179		
		R = 0.508, *R*^2^ = 0.258, Adjusted *R*^2^ = 0.210, *p* < 0.001; Δ*R*^2^ = 0.005, ΔF = 0.662, *p* = 0.418						
	4	(constant)	13.198		<0.001		187.337	208.179
		Age	−0.084	−0.059	0.543	1.154		
		Media viewing time	0.241	0.110	0.279	1.269		
		Media preference level	−0.228	−0.070	0.472	1.175		
		Perceptual biases	0.038	0.571	<0.001	1.664		
		Interaction effect 1	0.004	0.067	0.539	1.492		
		Interaction effect 2	−0.012	−0.169	0.264	2.829		
		Interaction effect 3	−0.007	−0.115	0.434	2.662		
		R = 0.513, *R*^2^ = 0.263, Adjusted *R*^2^ = 0.207, *p* < 0.001; Δ*R*^2^ = 0.005, ΔF = 0.617, *p* = 0.434						

Interaction effect 1 is represented by perceptual bias × media viewing time. Interaction effect 2 is represented by perceptual bias × media preference level. Interaction effect 3 is represented by perceptual bias × media viewing time × media preference level. AIC, Akaike’s information criterion. BIC, Bayesian information criterion. VIF, variance inflation factor.

**Table 5:** Summary of data for each group.

Group	Index	Age (years)	Media Viewing Time (hour)	Media Preference Level	Perceptual Biases (PSE; ms)	Manual Dexterity (Standard Score)
High manual dexterity group *n* = 71 male = 25 children female = 46 children	Mean	9.1	2.4	2.4	−4.3	13.3
	Standard deviation	1.9	1.2	0.8	33.8	1.7
	Range	6–12	0–5	0–3	−89.47–105.4	11–18
	Skewness	0.03	0.55	−1.14	−0.29	0.45
	Kurtosis	−1.09	−0.07	0.47	1.73	−0.32
Low manual dexterity group *n* = 29 male = 15 female = 14	Mean	9.3	2.3	2.3	−43.4	8.3
	Standard deviation	2.0	1.3	0.9	44.0	1.2
	Range	6–12	0–5	0–3	−150–88.84	6–10
	Skewness	−0.03	0.57	−1.34	0.45	−0.60
	Kurtosis	−1.43	−0.47	0.88	2.12	−0.48

PSE, point of subjective equality.

## Appendix A

**Table A1:** Raw data for the entire cohort.

No	Age (Years)	Sex	Preferred Hand (handedness)	Questionnaire On Media Viewing		TOJ Task	Manual Dexterity Test of the M-ABC-2		
				Media Viewing Time (Hour)	Media Preference Level	PSE	Component Score	Standard Score	Percentile
1	10	M	R	3	3	−44.73	35	13	84
2	8	F	L	2	2	−45.87	26	9	37
3	9	F	R	2	2	−1.653	39	15	95
4	10	F	R	2	2	−41.38	28	9	37
5	12	M	R	3	3	17.65	36	13	84
6	11	M	R	2	3	33.67	33.5	12	75
7	12	M	R	3	3	−24.72	24	8	25
8	8	F	L	2	3	66.88	36	13	84
9	9	F	R	2	3	−12.34	37.5	15	95
10	9	F	R	2	3	−89.45	31.5	11	63
11	9	M	R	5	3	−49.32	27	9	37
12	8	M	L	1	3	−39.67	27.5	9	37
13	12	F	R	2	2	−25.27	33.5	12	75
14	10	M	R	5	3	−2.319	36.5	14	91
15	7	F	R	3	3	−49.89	32	11	63
16	7	F	L	1	3	−1.758	23	7	16
17	12	M	R	3	3	−88.93	24	8	25
18	10	F	R	2	3	−80.35	28	9	37
19	9	M	L	3	2	2.523	36.5	14	91
20	12	F	R	2	3	−13.78	34.5	13	84
21	7	M	R	2	3	−74.58	31.5	11	63
22	12	F	R	5	3	25.24	38	15	95
23	8	M	R	1	3	−47.86	30.5	11	63
24	11	F	R	0	1	−5.713	31.5	11	63
25	12	M	R	2	3	2.132	40.5	17	99
26	11	M	R	5	3	−17.56	25	8	25
27	12	M	R	2	1	−2.593	37	14	91
28	12	F	R	4	2	−18.72	30.5	11	63
29	12	F	R	4	2	2.134	33.5	12	75
30	8	M	R	2	2	12.33	32	11	63
31	9	F	L	3	2	2.102	35	13	84
32	11	F	R	3	3	−42.5	35	13	84
33	8	F	R	3	3	17.2	32	11	63
34	10	M	R	1	0	12.69	29	10	50
35	6	M	R	3	3	13.74	34	12	75
36	11	M	R	4	1	−52.7	28	9	37
37	10	M	R	3	3	−25.17	35.5	13	84
38	11	M	R	2	3	−7.438	30.5	11	63
39	8	F	R	2	1	−41.25	35.5	13	84
40	8	F	L	1	3	25.67	38	15	95
41	10	F	R	2	0	−60.28	33.5	12	75
42	12	F	R	4	3	−28.57	24.5	8	25
43	12	F	R	2	2	−78.83	19	6	9
44	10	F	L	1	1	49.79	35	13	84
45	7	F	R	1	0	1.039	38	15	95
46	10	M	R	1	3	−5.977	36	13	84
47	9	M	R	2	3	−50.3	29.5	10	50
48	7	F	R	1	1	18.56	37.5	15	95
49	6	F	R	3	3	−150	24	8	25
50	12	F	R	4	3	−37.31	37	14	91
51	10	M	R	3	3	−89.47	34.5	13	84
52	10	F	R	1	1	8.513	23.5	8	25
53	8	F	R	2	2	−10.65	32	11	63
54	7	F	R	2	3	−80.35	21.5	7	16
55	12	M	R	1	3	−25.3	25.5	9	37
56	9	F	R	1	1	2.253	39.5	16	98
57	8	F	R	2	3	7.618	38.5	15	95
58	11	M	R	3	2	11.65	41.5	18	99.5
59	9	M	R	0	2	−54.62	23	7	16
60	8	F	R	4	2	−21.28	33.5	12	75
61	6	F	R	1	1	15.46	38	15	95
62	12	F	R	5	3	−75.15	34.5	13	84
63	9	M	L	1	2	5.086	32.5	12	75
64	7	M	R	2	3	−62.07	22	7	16
65	11	F	R	5	3	12.7	36.5	14	91
66	11	M	R	5	2	−26.77	27.5	9	37
67	9	F	R	2	3	36.22	35	13	84
68	8	F	L	3	2	−1.766	38	15	95
69	10	M	L	2	2	−25.29	31.5	11	63
70	7	M	R	1	3	3.581	36.5	14	91
71	7	F	R	1	3	105.4	35.5	13	84
72	10	F	R	5	3	24.74	38.5	15	95
73	10	F	R	2	3	13.79	36.5	14	91
74	8	M	R	3	3	−2.125	31	11	63
75	7	F	R	2	2	−7.782	37	14	91
76	6	F	R	1	3	−88.55	30	10	50
77	8	F	R	4	2	−5.67	32.5	12	75
78	11	F	R	1	1	−58.4	19.5	6	9
79	9	M	R	2	2	12.69	33.5	12	75
80	7	M	R	2	3	−7.35	33	12	75
81	6	F	R	2	3	17.1	38	15	95
82	7	M	R	3	3	−76.54	20	6	9
83	9	F	R	2	3	5.078	34.5	13	84
84	8	F	R	2	3	−2.126	41	17	99
85	10	F	R	2	2	−2.126	40	16	98
86	11	F	R	2	2	39.52	39.5	16	98
87	7	F	R	3	2	29.36	38	15	95
88	8	M	R	4	2	12.06	27.5	9	37
89	6	M	R	4	3	−84.35	34	12	75
90	7	F	L	3	3	−26.7	26	9	37
91	10	F	R	4	2	−11.33	39	15	95
92	8	F	R	2	3	88.84	26	9	37
93	12	F	R	2	2	−24.33	26.5	9	37
94	7	M	L	1	0	−107.7	30	10	50
95	9	M	R	2	3	−0.6862	37.5	15	95
96	6	F	R	1	3	−19.37	36.5	14	91
97	12	F	R	2	1	32.59	35.5	13	84
98	11	F	R	5	3	5.219	35	13	84
99	6	M	R	0	1	−1.004	34.5	13	84
100	7	F	R	2	3	1.952	32.5	12	75
Mean	9.2			2.4	2.4	−15.6	32.5	11.9	67.9
SD	1.9			1.2	0.8	41.1	5.4	2.7	26.7
Range	6–12			0–5	0–3	−150–105.4	19–41.5	6–18	9–99.5
Skewness	0.02			0.54	−1.20	−0.31	−0.66	−0.25	−0.74
Kurtosis	−1.20			−0.25	0.59	0.99	−0.38	−0.63	−0.74

TOJ, Temporal Order Judgment; PSE, Point of Subjective Equality; M-ABC-2, The Movement Assessment Battery for Children—2nd edition; M, Male; F, Female; R, Right; L, Left; SD, Standard deviation.
